# Neuronal Colocalization of μ-Opioid Receptor, κ-Opioid Receptor, and Oxytocin Receptor mRNA in the Central Nucleus of the Amygdala in Male and Female Mice

**DOI:** 10.1523/ENEURO.0059-25.2025

**Published:** 2025-09-05

**Authors:** Khalin E. Nisbett, George F. Koob

**Affiliations:** ^1^Graduate Program in Neuroscience, Graduate College, University of Illinois Chicago, Chicago, Illinois 60607; ^2^Neurobiology of Addiction Section, Integrative Neuroscience Research Branch, National Institute on Drug Abuse Intramural Research Program, National Institutes of Health, Baltimore, Maryland 21224

**Keywords:** central amygdala, coexpression, kappa, mu, opioid, oxytocin

## Abstract

Given the observed interaction and reports of oxytocin, μ-opioid receptor, or κ-opioid receptor expression in brain regions important to emotion regulation (i.e., the central amygdala), we hypothesized that oxytocin (*oxtr*), μ-opioid (*oprm1*), and κ-opioid (*oprk1*) receptor mRNA were colocalized to the same cells in the central amygdala. RNAscope in situ hybridization performed on fresh-frozen coronal brain sections was used to label cells containing *oxtr*, *oprm1*, and/or *oprk1*. The coronal sections were imaged using a 40× objective (widefield fluorescence) on a Leica Thunder fluorescent microscope, and the images were processed using open-source ImageJ/Fiji software and analyzed using the Imaris software. The central amygdala was identified using Paxinos and Watson's *The Mouse Brain in Stereotaxic Coordinates* ( Paxinos and Franklin, 2019). Eight distinct cell populations were enumerated (i.e., *oxtr*-only, *oprm1*-only, *oprk1*-only, *oxtr* + *oprm1*-only, *oxtr* + *oprk1*-only, *oprm1* + *oprk1*-only, *oxtr* + *oprm1* + *oprk1*, and nontranscript cells). Our findings demonstrated that 47% of cells in the central amygdala express *oxtr* with *oprm1* and/or *oprk1*. Of the *oxtr*-expressing cells, 38% colocalized only *oprm1*, and 56% of *oxtr*-expressing cells colocalized both *oprm1* and *oprk1*. However, 53% of *oprm1*-expressing cells colocalized *oxtr*, and 61% of *oprk1*-expressiong cells colocalized *oxtr*. These findings suggest that opioid and oxytocin receptors can function at the cellular level through morphological interactions. Future work will examine the physiological basis for the interaction between opioid and oxytocin receptors using transgenic behavior and electrophysiological assays.

## Significance Statement

We report novel findings that a large proportion of central amygdala cells coexpress *oprm1* and *oxtr* receptor mRNA, and a substantial proportion of central amygdala cells coexpress *oprm1*, *oxtr*, and *oprk1*. These data are significant and support our hypothesis of an interaction between opioid and oxytocin receptors, an interaction that has not yet been explored in the central nervous system. These findings underscore the unique and intricate role of opioids in regulating oxytocinergic systems, providing valuable insights into their therapeutic implications for anxiety disorders. This interaction is particularly interesting when considering that both systems may have efficacy in mitigating some neuroadaptations that are associated with substance use disorders, particularly alcohol misuse and alcohol use disorder, which are often comorbid with anxiety.

## Introduction

Neuropeptides have a broad range of functions related to brain health and are involved in complex behaviors. For example, opioids and oxytocin can have similar or opposing actions through their respective signaling cascades. Nonetheless, both are hypothesized to mediate parturition, lactation, nociception, social function, and emotion regulation (Douglas et al., 1995; Pasternak, 2018; Carter et al., 2020).

Many studies have shown that the activation of μ-opioid and oxytocin receptors has common effects. For example, oxytocin (Windle et al., 1997; Ring et al., 2006, 2010; Lukas and Neumann, 2014; Nisbett et al., 2023) and μ-opioid receptor agonists (Rezayof et al., 2009; Mutlu et al., 2012; Berrocoso et al., 2013; Samuels et al., 2017; Pekarskaya et al., 2021; Bruijnzeel et al., 2022) can reduce anxiety- and depression-like behavior and increase positive social interaction. Additionally, Yu et al. demonstrated that antinociceptive and analgesic effects of oxytocin can be blocked by μ- and κ-opioid receptor antagonists when administered in the lateral ventricles, periaqueductal gray, and nucleus accumbens (Ge et al., 2002; Gao and Yu, 2004; Gu and Yu, 2007).

However, early studies also demonstrated an antagonistic relationship between oxytocin and opioid systems in maternal behaviors, such as parturition and lactation (Douglas et al., 1995). Endogenous opioids and synthetic agonists that act on μ- and κ-opioid receptors have been shown to inhibit oxytocin release (Bicknell and Leng, 1982; Bondy et al., 1988; Zhao et al., 1988) and suppress the electrical activity of hypothalamic oxytocin neurons (Russell et al., 1989a,b; Inenaga et al., 1990). Complementary results showed that μ-opioid receptor antagonists increased oxytocin release (Bondy et al., 1988), activated oxytocin neurons (Pumford et al., 1993), and increased the rate of parturition and milk injection (Bicknell et al., 1988), whereas μ-opioid receptor agonists had opposite effects [i.e., delayed parturition (Russell et al., 1989a,b) and inhibited milk ejection (Clarke et al., 1979)]. Given that oxytocin is synthesized in the hypothalamus and pituitary gland and that antagonistic opioid actions were observed at the level of the hypothalamus, the aforementioned studies primarily support a hypothalamus-mediated antagonistic interaction between the oxytocin and opioid systems (Douglas et al., 1995; Nisbett, 2024).

Previous studies also demonstrated an antagonistic relationship between the oxytocin and μ-opioid systems with regard to anxiety, social attention, and appetite regulation. μ-Opioid receptor agonists can block oxytocin function, and μ-opioid receptor antagonists can potentiate oxytocin actions. In a recent study, we showed that blocking the endogenous μ-opioid receptor system with naloxone or CTAP (D-Phe-Cys-Tyr-D-Trp-Arg-Thr-Pen-Thr-NH2) can potentiate the anxiolytic-like effect of oxytocin (Nisbett et al., 2024). We also found that blocking the endogenous κ-opioid receptor system had opposite effects and blocked the anxiolytic-like effect of oxytocin. Others demonstrated that blocking opioid receptors with naltrexone while simultaneously administering oxytocin enhanced prolonged and selective social attention in monkeys (Dal Monte et al., 2017). In another study, naltrexone and oxytocin synergistically reduced sucrose intake in rats (Head et al., 2021). Given that anxiety, social attention, appetite regulation, and pain are primarily mediated by extrahypothalamic brain regions, such as the central nucleus of the amygdala (central amygdala), these latter findings suggest the additional hypothesis of an extrahypothalamic interaction between the opioid and oxytocin systems.

Altogether, reports of antagonistic relationships between the opioid and oxytocin systems indicate that interactions between both systems may occur at the level of the hypothalamus (where oxytocin is synthesized) and in extrahypothalamic brain regions that express oxytocin receptors (Nisbett, 2024). One hypothesis to explain such interactions is that μ- and κ-opioid receptors are expressed near oxytocin receptors to influence oxytocin function.

The central amygdala is particularly interesting because of its importance in emotion regulation, sociality, appetite regulation, and pain. Mansour et al. reported κ- but not μ-opioid receptor binding in the central amygdala (Mansour et al., 1987, 1988). However, other studies exhibited moderate to dense μ-opioid receptor mRNA and protein expression in the central amygdala (Mansour et al., 1994; Ding et al., 1996; Zhang et al., 2015). Numerous studies also reported dense expression of oxytocin receptors and oxytocin binding sites in the central amygdala (Veinante and Freund-Mercier, 1997; Huber et al., 2005).

In the present study, we used RNAscope in situ hybridization to investigate the localization of oxytocin receptor mRNA (*oxtr*) and μ-opioid receptor mRNA (*oprm1*) in the central amygdala. Based on extensive neuropharmacological data that suggest functional interactions between μ-opioid receptor and oxytocin systems, we hypothesized that *oxtr* and *oprm1* would show neuronal colocalization. We also assessed κ-opioid receptor mRNA (*oprk1*) localization to explore the morphological relationship of κ-opioid receptors relative to μ-opioid and oxytocin receptors.

## Materials and Methods

### Subjects

A total of 24,857 cells from 19 coronal sections from five C57Bl/6J mice (Jackson Laboratory) were used (three males, 8–12 weeks old and weighing 24–28 g, and two females, 8–12 weeks old and weighing 18–20 g, on the date of sacrifice). The mice were acclimated for at least 1 week before sacrifice. The mice were housed in same-sex groups (four per cage) in plastic cages (28 cm width × 17 cm length × 12 cm height) with *ad libitum* access to food and water. The mice were kept in a room with a 12/12 h light/dark cycle (lights on at 7 A.M.) with controlled temperature (22 ± 2°C) and humidity (50–60%). The mice were sacrificed during the light cycle.

### Tissue preparation: killing and cryosectioning

**Figure 1.**
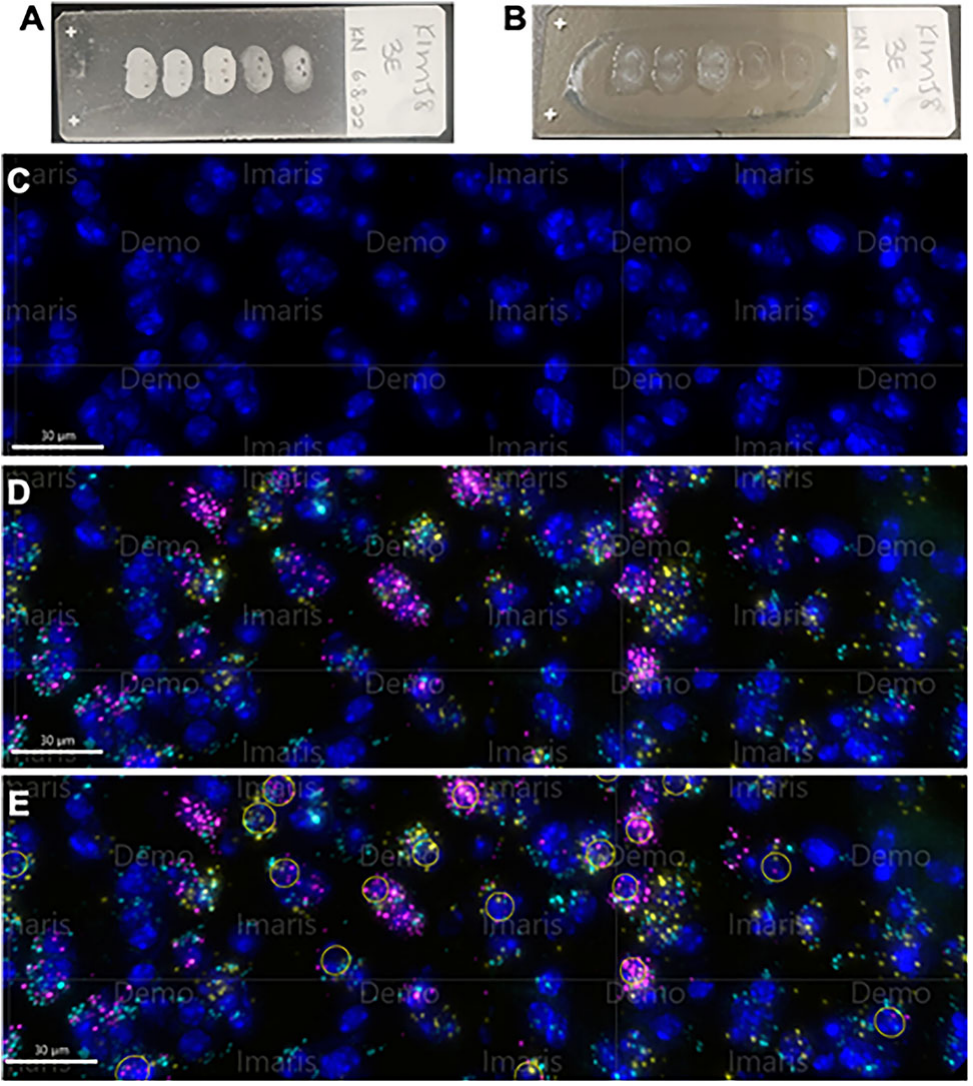
Tissue preparation and analysis. ***A***, The cryosectioned coronal tissue was collected on electrostatic microscope slides (Fisherbrand Superfrost Plus Microscope Slides, Thermo Fisher Scientific) and stored at −80°C. ***B***, A hydrophobic barrier (blue oval) was drawn on the microscope slides around the coronal section on the day of assaying. This allowed the reagents to be applied directly to the microscope slide for better control and waste management. After the assay was completed, the tissue was imaged and processed. ***C–E***, Illustrative images are from Imaris. DAPI stain (blue), *oprm1* (cyan), *oxtr* (yellow), and *oprk1* (pink). DAPI-stained cells without (***C***) and with (***D***) all puncta present. Imaris was used to determine nonexclusionary cell populations, such as “DAPI × *oprm1* × *oprk1* spots” (***E***) from which exclusionary cell populations (e.g., “*oprm1* + *oprk1*-only cells” and “*oprm1* + *oxtr* + *oprk1*-only cells”) were derived.

Mice were euthanized via isoflurane saturation followed by cervical dislocation. Isoflurane was obtained from Covetrus. Brains were then extracted and immediately flash-frozen in isopentane (Millipore Sigma), chilled to −65°C on dry ice, and stored at −80°C for 24–72 h. The brains were then cryosectioned in the coronal plane in the rostrocaudal direction into 50-mm-thick sections, starting at the frontal association cortex and anterior olfactory area (i.e., 3.05 anterior to the bregma) until the anterior commissure separated into three distinct structures (i.e., at bregma). Cryosectioning was guided by a mouse brain atlas (Paxinos and Franklin, 2019). Starting at bregma, the brains were sectioned into 20-mm-thick sections and collected on Fisherbrand Superfrost Plus Microscope Slides (Thermo Fisher Scientific). Ten replicates of 20 µm sections were collected; replicates 1–4 were collected, while sets 5–10 were not collected. This strategy yielded 4–5 central amygdala sections that were 200 µm apart. Thus, each microscope slide was representative of the entire central amygdala (Fig. 1*A*). Slides were stored at −20°C during tissue collection and then at −80°C until further analysis. Brain sections were stored at −80°C for up to 14 d before in situ hybridization.

### In situ hybridization and tissue preservation

In situ hybridization was performed using the RNAscope Multiplex Fluorescent Detection Kit (catalog #323110, Advanced Cell Diagnostics) according to the manufacturer's instructions (Wang et al., 2015). The fresh-frozen sections were fixed in chilled 10% formalin (4°C) for 15 min, rinsed with 1× PB-DEPC (i.e., 10× phosphate buffer solution; catalog #BP39920, Thermo Fisher Scientific) that was diluted with RNAse inhibition diethyl polycarbonate treated water (catalog #693520, Millipore Sigma), and fixed with increasing concentrations (50, 70, and 100%, diluted with double-distilled water) of anhydrous ethanol (The Warner Graham Company). Slides were stored overnight in 100% ethanol at −20°C. The next morning, the slides were allowed to dry in the open air. After drawing a hydrophobic barrier using an ImmEdge Pen (catalog #H-4000; Vector Laboratories) around coronal sections of the microscope slide (Fig. 1*B*), they were treated with hydrogen peroxide and protease pretreat-4 for protease digestion. We hybridized the sections with probes against *oprm1*, *oxtr*, and *oprk1* mRNA (catalog #315841, 412171, and 316111, respectively, Advanced Cell Diagnostics) for 2 h at 40°C. Probes were amplified using RNAscope amplifiers, serially tagged with horseradish peroxidase, and labeled with opal dyes at 1:750 dilution. Although the RNAscope probes have a commercial origin, they are the standard probes synthesized by ACDBio and have receptor-specific activity. These probes have also been utilized in prior studies (Han et al., 2022; Holland et al., 2023; Li et al., 2024). Peroxidase activity was blocked, and excess reagents were washed before developing the next probe. All reagents except the diluted opal dyes were from the RNAscope Multiplex Fluorescent Detection Kit. The following opal dyes from Akoya Biosciences were used: Opal 520 (catalog #FP1487001KT), Opal 620 (catalog #FP1495001KT), and Opal 690 (catalog #FP1497001KT). After all probes were tagged with the opal dyes, the coronal sections were washed in phosphate-buffered diethyl pyrocarbonate-treated water (PB-DEPC) and coverslipped with Fluoromount-G 4′,6-diamidino-2-phenylindole (DAPI; catalog #17984-24, Electron Microscopy Science). The coverslip was secured using clear nail polish (catalog #72180, Electron Microscopy Science), and the slides were allowed to dry overnight. Preservation and drying occurred under a chemical fume hood.

### Tissue processing and analysis

The coronal sections were mapped using a 5× objective (bright field) and imaged using a 40× objective (widefield fluorescence) on a Thunder Fluorescent Microscope (Leica Microsystems). The images were processed using open-source ImageJ/Fiji software and analyzed using Imaris 9.9.1 software (Bitplane). Regions of interest [capsular (CeC), lateral (CeL), and medial (CeM) nuclei of the central amygdala] were drawn according to a mouse brain atlas (Paxinos and Franklin, 2019). DAPI-stained nuclei and fluorescent mRNA puncta were labeled using the Imaris spots tool and manually edited to more accurately localize DAPI-stained nuclei (DAPI spots) and mRNA puncta (see Fig. 1 for sample images). DAPI spots that colocalized with a specified puncta marker were filtered to create nonexclusionary cell populations. Puncta within 7.5 mm of the spot center were considered to be colocalized. For example, all DAPI spots that colocalized with *oprm1* puncta were filtered to create a new population: “DAPI × *oprm1* spots.” This nonexclusionary cell population refers to all *oprm1*-expressing cells regardless of whether or not they express *oxtr* and/or *oprk1*. Exclusionary cell populations refer to cells that express only the mRNA identified in their population label. Numerical data (i.e., the various spot populations) were exported to Microsoft Excel to determine exclusionary cell populations (e.g., DAPI spots that colocalized with *oprm1* but not *oprk1* or *oxtr*; i.e., “*oprm1*-only cells”) and calculate cell distributions across each identified region and section.

### Statistical analysis

Statistical analyses were conducted using Prism software (GraphPad). One-way analyses of variance (ANOVAs) were used throughout the study to determine differences in cell phenotype proportions across the central amygdala subdivisions and along the rostrocaudal axis. Data analysis includes cells that do not express any of the mRNA studied (i.e., no-transcript cells). ANOVAs that yielded a significant main effect of treatment were further analyzed using Holm–Sidak's multiple-comparison post hoc test. Values of *p* ≤ 0.05 were considered statistically significant. Data are expressed as the mean ± standard error of the mean.

## Results

### Relative expression of *oprm1*, *oxtr*, and *oprk1* in the central amygdala

**Figure 2.**
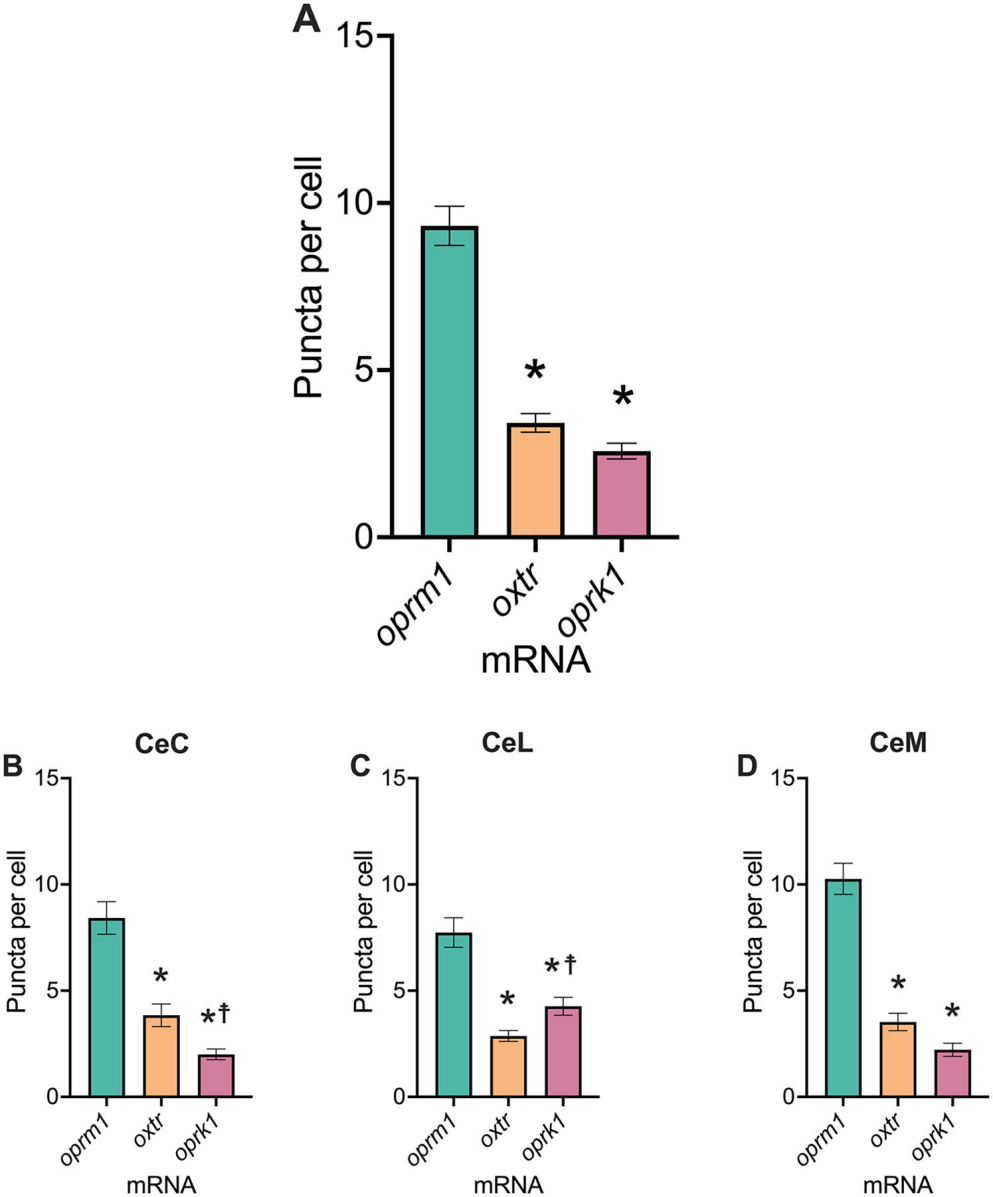
mRNA density throughout the central amygdala. The density of *oprm1* puncta was higher than *oxtr* and *oprk1* puncta. The number of puncta per cell for each mRNA was the following: central amygdala [*oprm1*, 9.3 ± 0.6; *oxtr*, 3.4 ± 0.3; *oprk1*, 2.6 ± 1.0 (one-way ANOVA, *F*_(2,54)_ = 84.62; *p* < 0.0001; *oprm1* vs *oxtr*, *p* < 0.0001; *oprm1* vs *oprk1*, *p* < 0.0001; *oxtr* vs *oprk1*, *p* = 0.1415)] (***A***), CeC [*oprm1*, 8.4 ± 0.8; *oxtr*, 3.8 ± 0.5; *oprk1*, 2.0 ± 0.3 (one-way ANOVA, *F*_(2,54)_ = 35.14; *p* < 0.0001; *oprm1* vs *oxtr*, *p* < 0.0001; *oprm1* vs *oprk1*, *p* < 0.0001; *oxtr* vs *oprk1*, *p* = 0.0233)] (***B***), CeL [*oprm1*, 7.7 ± 0.7; *oxtr*, 2.9 ± 0.3; *oprk1*, 4.3 ± 0.4 (one-way ANOVA, *F*_(2,42)_ = 26.09; *p* < 0.0001; *oprm1* vs *oxtr*, *p* < 0.0001; *oprm1* vs *oprk1*, *p* < 0.0001; *oxtr* vs *oprk1*, *p* = 0.0500)] (***C***), CeM [*oprm1*, 10.3 ± 0.7; *oxtr*, 3.5 ± 0.4; *oprk1*, 2.2 ± 0.3 (one-way ANOVA, *F*_(2,54)_ = 69.47; *p* < 0.0001; *oprm1* vs *oxtr*, *p* < 0.0001; *oprm1* vs *oprk1*, *p* < 0.0001; *oxtr* vs *oprk1*, *p* = 0.0782)] (***D***). *p *≤* 0.05, relative density of *oprm1* puncta compared with other puncta; ^☨^*p* < 0.05, relative density of *oprk1* puncta compared with other puncta.

We quantified mRNA puncta for *oprm1*, *oxtr*, and *oprk1* across the central amygdala by calculating puncta per total cell count. The density of *oprm1* puncta was ∼1.7-fold higher than *oxtr* and 2.7-fold higher than *oprk1* (Fig. 2*A*). *oxtr* and *oprk1* puncta densities were not significantly different. The mRNA densities were also significantly different in the three subdivisions of the central amygdala (i.e., CeC, CeM, and CeL; Fig. 2*B*–*D*). The statistical analysis showed that the *oprm1* density was higher than the *oxtr* and *oprk1* densities in each central amygdala subdivision. Only in the CeC and CeL were *oxtr* puncta significantly different from *oprk1* puncta. In the CeC, the *oxtr* puncta density was higher than *oprk1* puncta density. In the CeL, the *oprk1* puncta density was higher than *oxtr* puncta density. In the CeM, we observed a trend toward a higher density of *oxtr* puncta compared with *oprk1* puncta.

These three mRNA (*oprm1*, *oxtr*, and *oprk1*) were coexpressed in different combinations in central amygdala cells. As such, we identified eight cell types (Table 1).

**Table 1.** Cell-type nomenclature

Cell type	Expression		
	*oprm1*	*oxtr*	*oprk1*
*oprm1* + *oxtr* only	+	+	−
*oprm1* + *oxtr* + *oprk1*	+	+	+
*oprm1* only	+	−	−
*oxtr* only	−	+	−
*oprm1* + *oprk1* only	+	−	+
*oxtr* + *oprk1* only	−	+	+
*oprk1* only	−	−	+
No transcript	−	−	−

+ mRNA expression of respective receptor mRNA is positive in the specified cell type; ^−^mRNA expression of respective receptor mRNA is negative in the specified cell type.

The population of each cell type in the central amygdala and across each subdivision (CeC, CeL, and CeM) is reported in Table 2. Below, the data are reported as proportions of total cell populations in the central amygdala (CeC, CeL, and CeM). Given the differences in mRNA density, we also discuss the data in nonexclusionary subsets (i.e., subpopulations of *oprm1*-, *oxtr*-, and *oprk1*-expressing cells). Populations of nonexclusionary subsets are reported in Table 2, bottom.

**Table 2.** Populations of exclusionary cell types and nonexclusionary subsets

Cell type	Central amygdala	CeC	CeL	CeM
Exclusionary cell populations				
* oprm1* *+* *oxtr-*only	6,529	2,796	1,233	2,500
* oprm1* *+* *oxtr* *+* *oprk1*	4,440	1,087	1,720	1,633
* oprm1-*only	7,029	2,421	1,635	2,973
* oxtr-*only	641	217	105	319
* oprm1* *+* *oprk1-*only	2,647	846	690	1,111
* oxtr* *+* *oprk1-*only	197	31	81	85
* oprk1-*only	284	106	104	74
* *no transcript	3,090	1,175	565	1, 350
Nonexclusionary cell populations (subsets)				
* *All *oprm1*-expressing cells	20,645	7,150	5,278	8,217
* *All *oxtr*-expressing cells	11,807	4,131	3,139	4,537
* *All *oprk1*-expressing cells	7,568	2,070	2,595	2,903
* *Total cell count	24,857	8,679	6,133	10,045

### Distribution of each cell type throughout the central amygdala

**Figure 3.**
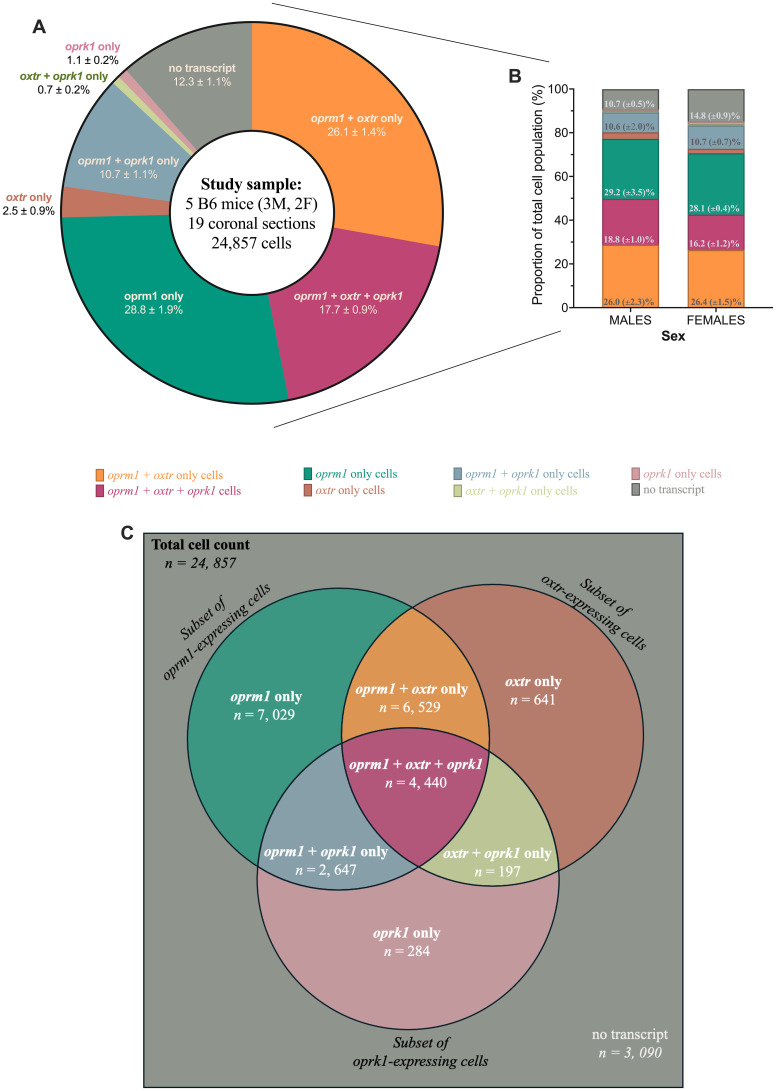
A large proportion of cells in the central amygdala are positive for *oprm1* and *oxtr*. ***A***, The donut chart shows the proportion of central amygdala cells that expressed *oprm1*, *oxtr*, and *oprk1* across five naive B6 mice. The proportion of each cell type throughout the central amygdala was the following: *oprm1* + *oxtr*-only (26.1 ± 1.4%), *oprm1* + *oxtr* + *oprk1* (17.7 ± 0.9%), *oprm1-*only (28.8 ± 1.9%), *oxtr*-only (2.5 ± 0.9%), *oprm1* + *oprk1*-only (10.7 ± 1.1%), *oxtr* + *oprk1*-only (0.7 ± 0.2%), *oprk1*-only (1.1 ± 0.2%), and no transcript (12.3 ± 1.1%). ***B***, The bar chart shows the same cell proportions as a factor of sex (i.e., two males vs three females). Proportions of cell types not listed were the following: males (*oxtr*-only cells comprised 2.9 ± 1.6%, *oxtr* + *oprk1* cells comprised 0.8 ± 0.3%, and *oprk1*-only cells comprised 1.0 ± 0.3% of the total central amygdala cell population) and females (*oxtr-*only cells comprised 2.1 ± 0.3%, *oxtr* + *oprk1*-only cells comprised 0.7 ± 0.2%, and *oprk1*-only cells comprised 1.2 ± 0.3% of the total central amygdala cell population). Experimental male (*n* = 3) and female (*n* = 2) groups were underpowered for sex-based statistical comparison. ***C***, The Venn diagram shows the population of each cell type within subsets of *oprm1*-, *oxtr*-, and *oprk1*-expressing cells. Cell-type proportions are reported as the mean ± standard error of the mean, representing an average of cell-type proportions of each section that was analyzed. Cell-type proportions for each section were calculated by dividing the number of cells of a particular cell type (e.g., *oxtr*-only cells, *oxtr* +*oprk1*-only cells, and no transcript) by the total cell count for that coronal section. See Extended Data Figure 3-1 for a distribution of each cell types across the experimental animal used in this study.

Almost half (∼43.8%) of the cells in the central amygdala expressed *oprm1* and *oxtr*, regardless of whether they also expressed *oprk1* (Fig. 3*A*). We also observed a similar distribution of cells in males and females (Fig. 3*B*). Proportion differences between males and females were <2.6% for all cell types except no-transcript cells, for which the difference was 4.1%. Female samples contained more no-transcript cells (14.8 ± 0.9%) than males (10.7 ± 0.5%). The distribution of each cell type for each animal is also shown in Extended Data Figure 3-1.

10.1523/ENEURO.0059-25.2025.f3-1 Figure 3-1 The distribution of various cell types across each experimental animal is similar. Download Figure 3-1, TIF file.

### Distribution of significant cell types across central amygdala subdivisions (CeC, CeL, and CeM) and the rostrocaudal axis

**Figure 4.**
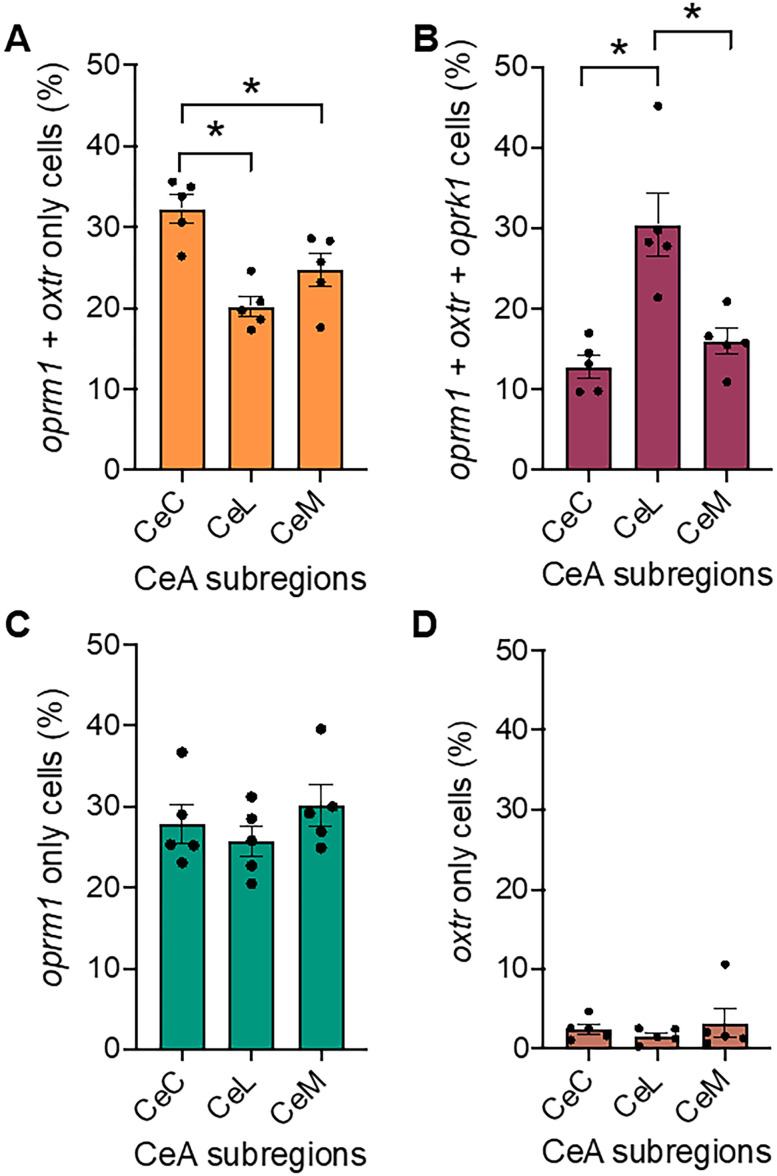
The proportion of cells that contained *oxtr* and *oprm1* changed significantly across central amygdala subdivisions. The figure shows differences in cell-type proportions across central amygdala subdivisions for the three most abundant cell types. ***A***, *oprm1* + *oxtr*-only cells comprised 32.3 ± 1.7% of cells in the CeC, 20.2 ± 1.2% of cells in the CeL, and 24.7 ± 2.0% of cells in the CeM (one-way ANOVA, *F*_(2,12)_ = 13.09; *p* = 0.0010; CeC vs CeL, *p* = 0.0008; CeC vs CeM, *p* = 0.0157; CeL vs CeM, *p* = 0.0850). ***B***, *oprm1* + *oxtr* + *oprk1* cells comprised 12.8 ± 1.4% of the CeC, 30.5 ± 3.9% of the CeL, and 15.9 ± 1.6% of the CeM (one-way ANOVA, *F*_(2,12)_ = 13.25; *p* = 0.0009; CeC vs CeL, *p* = 0.0013; CeC vs CeM, *p* = 0.4134; CeL vs CeM, *p* = 0.0037). ***C***, *oprm1-*only cells comprised 27.9 ± 2.4% of the CeC, 25.7 ± 1.9% of the CeL, and 30.1 ± 2.5% of the CeM (one-way ANOVA, *F*_(2,12)_ = 0.9046; *p* = 0.4306). ***D***, *oxtr*-only cells comprised 2.5 ± 0.6% of the CeC, 1.6 ± 0.4% of the CeL, and 3.3 ± 1.8% of the CeM (one-way ANOVA, *F*_(2,12)_ = 0.4953; *p* = 0.6213). Cell-type proportions are reported as the mean ± standard error of the mean, representing an average of cell-type proportions for central amygdala subdivisions in each coronal section. Cell-type proportions for each central subdivision were calculated by dividing the number of cells of a particular type (e.g., *oxtr* + *oprk1*-only, no transcript, and *oxtr*-only) by the total cell count for the particular subdivision within each coronal section. *p < 0.05, relative to CeC; ^☨^*p* < 0.05, relative to CeL.

**Figure 5.**
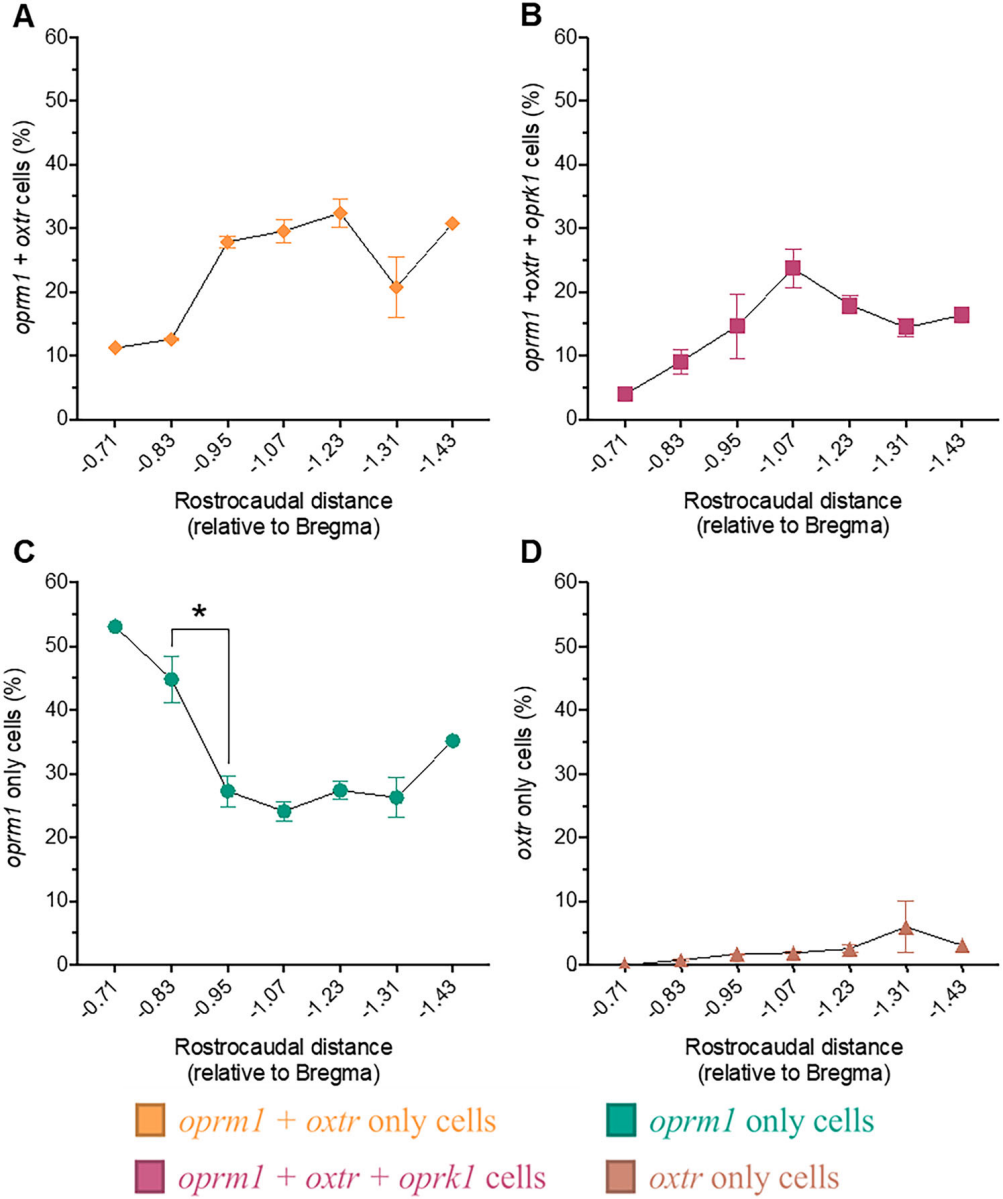
The distribution of *oprm1* *+* *oxtr*-only cells and *oprm1*-only cells changed significantly across the rostrocaudal axis throughout the central amygdala. The figure shows the topographical distribution of *oprm1* *+* *oxtr*-only (***A***), *oprm1* *+* *oxtr* *+* *oprk1* (***B***), *oprm1*-only (***C***), and *oxtr*-only (***D***) cells across the rostrocaudal axis in the central amygdala. *oprm1* *+* *oxtr*-only cells ranged from 7.6 to 35.7%. *oprm1* *+* *oxtr* *+* *oprk1* cells ranged from 3.9 to 30.7%. *oprm1*-only cells ranged from 17.6 to 53.1%. *oxtr*-only cells ranged from 0.0 to 18.1%. A trend toward an increase from 12.6 ± 0.2% to 27.9 ± 0.9% was observed for *oprm1* *+* *oxtr*-only cells (***A***; one-way ANOVA, *F*_(6,12)_ = 2.725; *p* = 0.0259; −0.83 vs −0.95; *p* = 0.0627), and a decrease from 44.9 ± 3.7% to 27.3 ± 2.4% was observed for *oprm1*-only cells (***C***; one-way ANOVA, *F*_(6,12)_ = 10.58; *p* = 0.0003; −0.83 vs −0.95; *p* = 0.0062) from −0.83 to −0.95 relative to the bregma. No significant changes across the rostrocaudal axis were observed for *oprm1* *+* *oxtr* *+* *oprk1* cells (one-way ANOVA, *F*_(6,12)_ = 2.985; *p* = 0.0506) or *oxtr*-only cells (one-way ANOVA, *F*_(6,9)_ = 0.6341; *p* = 0.7015). Cell-type proportions are reported as the mean ± standard error of the mean, representing an average of cell-type proportions across the rostrocaudal coordinate. Each coronal section aligns with a particular rostrocaudal axis coordinate. Cell-type proportions at each rostrocaudal axis coordinate were calculated by dividing the number of cells of a particular type (e.g., *oxtr*-only, *oxtr* + *oprk1*, and no transcript) by the total cell count at the particular rostrocaudal axis coordinate. *p < 0.05, compared with rostrocaudal distance: −0.83 relative to bregma.

**Figure 6.**
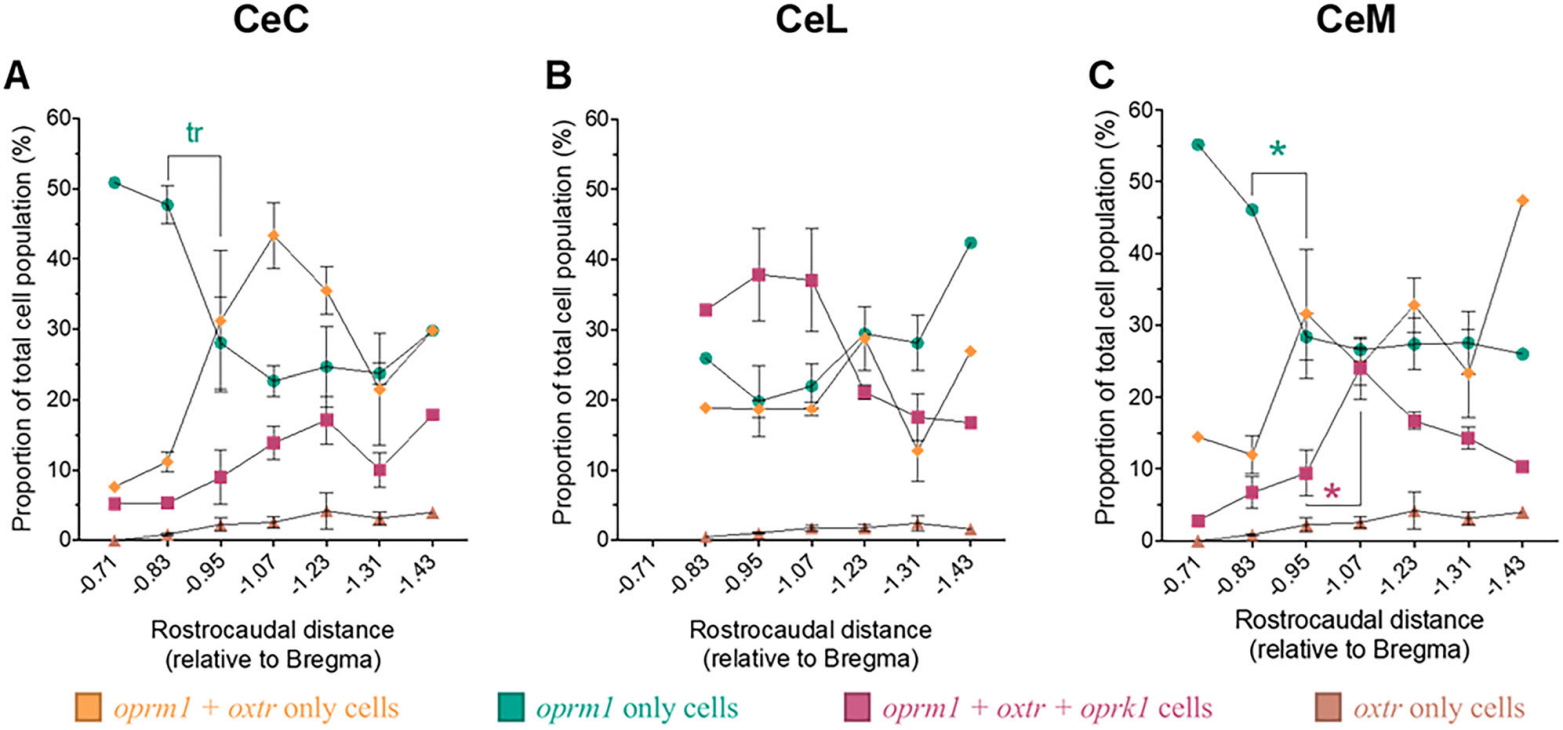
The distribution of *oprm1*-only and *oprm1* + *oxtr* + *oprk1* cells changed significantly across the rostrocaudal axis of the CeM. ***A***, In the CeC, we observed a trend toward a decrease from 47.7 ± 2.7% to 28.1 ± 6.5% for *oprm1*-only cells from −0.83 to −0.95 (one-way ANOVA, *F*_(6,12)_ = 5.304; *p* = 0.0069; −0.83 vs −0.95; *p* = 0.0554). No significant changes were observed across the rostrocaudal axis for *oprm1* *+* *oxtr*-only cells (one-way ANOVA, *F*_(6,12)_ = 2.676; *p* = 0.0693), *oprm1* *+* *oxtr* *+* *oprk1* cells (one-way ANOVA, *F*_(6,12)_ = 1.796; *p* = 0.1826), or *oxtr*-only cells (one-way ANOVA, *F*_(6,12)_ = 0.7048; *p* = 0.6521). ***B***, In the CeL, no significant changes were observed across the rostrocaudal axis for *oprm1* *+* *oxtr*-only cells (one-way ANOVA, *F*_(5,9)_ = 3.020; *p* = 0.0714), *oprm1* *+* *oxtr* *+* *oprk1* cells (one-way ANOVA, *F*_(5,9)_ = 1.761; *p* = 0.2171), *oprm1*-only cells (one-way ANOVA, *F*_(5,9)_ = 2.478; *p* = 0.1121), or *oxtr*-only cells (one-way ANOVA, *F*_(5,9)_ = 0.5899; *p* = 0.7088). Note that the CeL was not present at anterior/posterior coordinate −0.71. ***C***, In the CeM, a significant increase from 9.5 ± 3.2% to 24.1 ± 4.3% for *oprm1* *+* *oxtr* *+* *oprk1* cells from −0.95 to −1.07 (one-way ANOVA, *F*_(6,12)_ = 3.403; *p* = 0.0337; −0.95 vs −1.07; *p* = 0.0490) and a significant decrease from 46.1 ± 0.7% to 28.4 ± 3.3% for *oprm1*-only cells from −0.83 to −0.95 (one-way ANOVA, *F*_(6,12)_ = 6.026; *p* = 0.0042; −0.83 vs −0.95; *p* = 0.0395) were also observed. No significant changes were observed across the rostrocaudal axis for *oprm1* *+* *oxtr*-only cells (one-way ANOVA, *F*_(6,12)_ = 2.161; *p* = 0.5193) or *oxtr*-only cells (one-way ANOVA, *F*_(6,12)_ = 0.5474; *p* = 0.7633). All coordinates cited are relative to bregma. *p < 0.05; compared with rostrocaudal distance, −0.95 relative to bregma. See Extended Data Figure 6-1 for distribution of each cell type across the rostrocaudal axis in the central amygdala and each subdivision.

We also analyzed changes in the proportion of each cell type across the three subdivisions of the central amygdala (i.e., CeC, CeL, and CeM) and across the rostrocaudal axis (Figs. 4–6). We found that *oprm1* + *oxtr*-only, *oprm1* + *oxtr* + *oprk1*, and *oprm1*-only cells comprised most of the total cell population and changed significantly across central amygdala subdivisions (Fig. 4) and the rostrocaudal axis (Fig. 5). The *oxtr*-only cells were also included as a control.

10.1523/ENEURO.0059-25.2025.f6-1 Figure 6-1 **Distribution of each cell type across the rostrocaudal axis in the central amygdala (A) and each subdivision: CeC (B), CeL (C), and CeM (D).** In the central amygdala, the mean cell distribution ranges were the following: *oprm1 + oxtr*-only (7.6-35.7%), *oprm1 + oxtr + oprk1* (3.9-30.7%), *oprm1*-only (17.6-53.1%), *oxtr*-only (0-18.1%), *oprm1 + oprk1*-only (5.4-24.5%), *oxtr + oprk1* (0.1-4.7%), *oprk1*-only (0.3-4.4%), no transcript (5.8-20.8%) (**A**). In the CeC, the mean cell distribution ranges were the following *oprm1 + oxtr*-only (7.6-40.9%), *oprm1 + oxtr + oprk1* (5.2-17.9%), *oprm1*-only (21.6-50.9%), *oxtr*-only (0-4.0%), *oprm1 + oprk1*-only (6.0-15.1%), *oxtr + oprk1* (0.0-1.1%), *oprk1*-only (0.3-2.6%), no transcript (7.8-20.6%) (**B**). In the CeL, the mean cell distribution ranges were the following: *oprm1 + oxtr*-only (6.1-37.0%), *oprm1 + oxtr + oprk1* (12.4-55.7%), *oprm1*-only (14.8-42.4%), *oxtr*-only (0.3-4.0%), *oprm1 + oprk1*-only (4.7-27.8%), *oxtr + oprk1* (0.0-5.8%), *oprk1*-only (0.0-9.4%), no transcript (2.9-17.1%). Note that the CeL was not present at anterior/posterior coordinate -0.71 relative to bregma (**C**). In the CeM, the mean cell distribution ranges were the follow: *oprm1 + oxtr*-only (12.0-47.4%), *oprm1 + oxtr + oprk1* (2.8-20.2%), *oprm1*-only (24.1-55.2%), *oxtr*-only (0.0-1.7%), *oprm1 + oprk1*-only (0.0-15.4%), *oxtr + oprk1* (0.0-0.6%), *oprk1*-only (0.3-1.0%), no transcript (9.7-19.8% (**D**). Download Figure 6-1, TIF file.

### Distribution of significant cell types across central amygdala subdivisions

The proportion of the total cell population comprising *oprm1* *+* *oxtr*-only, and *oprm1* *+* *oxtr* *+* *oprk1* cells differed across central amygdala subdivisions (Fig. 4). The *oprm1* *+* *oxtr*-only cell proportion was higher in the CeC than in the CeL and CeM. However, no difference was observed between the CeL and CeM. The *oprm1* *+* *oxtr* *+* *oprk1* cell proportion was higher in the CeL compared with that in the CeC and CeM, with no difference between the CeC and CeM. The proportion of *oprm1*-only and *oxtr*-only cells did not differ across central amygdala cell subdivisions. No other significant differences between central amygdala subregions were found for the remaining cell types.

### Distribution of significant cell types across the rostrocaudal axis: central amygdala

The distribution of *oprm1*-only cells changed across the rostrocaudal axis in the central amygdala (Fig. 5). Here, we observed an 18% decrease in *oprm1*-only cells (*p* = 0.0062; from −0.83 to −0.95 relative to bregma; Fig. 5*C*). Although not significant, a trend was found for an increase in *oprm1* *+* *oxtr*-only cells (*p* = 0.0627; i.e., the proportion of *oprm1* *+* *oxtr*-only cells increased by 15% from −0.83 to −0.95 relative to bregma; Fig. 5*A*). No significant changes were observed in the *oxtr* + *oprm1*-only, *oprm1* *+* *oxtr* *+* *oprk1*, or *oxtr*-only cell distributions across the rostrocaudal axis.

The distribution of *oprm1*-only and *oprm1* *+* *oxtr* *+* *oprk1* cells also changed across the rostrocaudal axis in the CeC and CeM but not in the CeL (Fig. 6). Extended Data Figure 6-1 also demonstrates the distribution of each cell type across the rostrocaudal axis in the central amygdala and each subdivision.

### Distribution of significant cell types across the rostrocaudal axis: CeC

Proportions of *oprm1*-only cells in the CeC changed significantly across the rostrocaudal axis (Fig. 6*A*). Our analysis demonstrated a trend toward a 20% decrease (*p* = 0.0554) in *oprm1* cell proportions from −0.83 to −0.95 relative to bregma. We did not observe significant changes across the rostrocaudal axis for *oprm1* *+* *oxtr-*only, *oprm1* *+* *oxtr* *+* *oprk1*, or *oxtr-*only cells.

### Distribution of significant cell types across the rostrocaudal axis: CeM

Proportions of *oprm1* *+* *oxtr* *+* *oprk1* and *oprm1*-only cells in the CeM also changed significantly across the rostrocaudal axis (Fig. 6*B*). We observed a 15% increase (*p* = 0.0490) in *oprm1* *+* *oxtr* *+* *oprk1* cells from −0.95 to −1.07 relative to bregma and an 18% decrease (*p* = 0.0395) in the *oprm1*-only cell proportion from −0.83 to −0.95 (relative to bregma).

**Figure 7.**
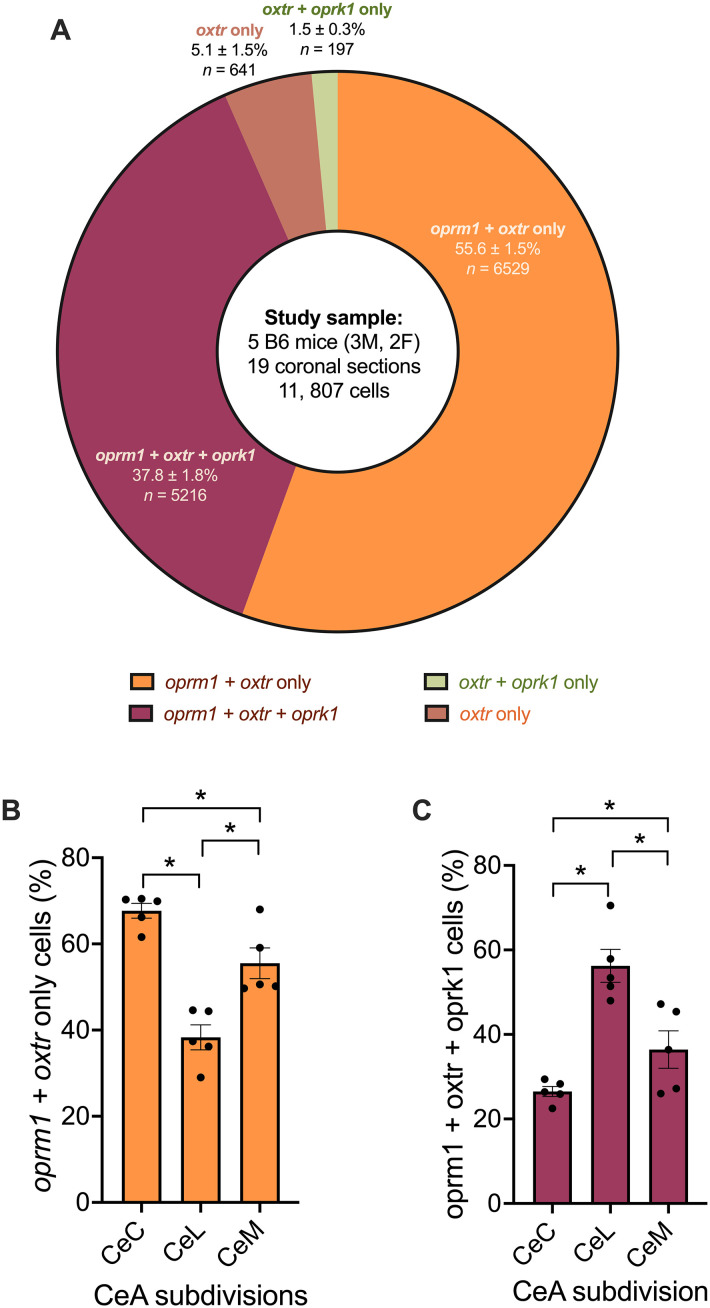
A majority of *oxtr*-expressing cells also express *oprm1*. ***A***, Cell-type distributions were different across the CeC, CeL, and CeM. Of *oxtr*-expressing cells throughout the central amygdala, 55.6 ± 1.5% expressed only *oprm1* (*oprm1* *+* *oxtr* cells), 37.8 ± 1.8% expressed both *oprm1* and *oprk1* (*oprm1* *+* *oxtr* *+* *oprk1* cells), 1.5 ± 0.3% expressed only *oprk1* (*oxtr* *+* *oprk1* cells), and 5.1 ± 1.5% expressed no other transcript (i.e., *oxtr*-only cells). ***B***, *oprm1* *+* *oxtr*-only cell distributions across central amygdala cell divisions were the following CeC (67.7 ± 1.7%), CeL (38.3 ± 2.9%), CeM (55.5 ± 3.6%). ***C***, *oprm1* *+* *oxtr* *+* *oprk1* cell distributions across central amygdala cell divisions were the following: CeC (26.5 ± 1.2%), CeL (56.3 ± 3.9%), CeM (36.5 ± 4.4%). Significant differences between central amygdala subdivisions were observed for *oprm1* *+* *oxtr*-only cells (one-way ANOVA, *F*_(2,12)_ = 27.10; *p* < 0.0001; CeC vs CeL, *p* < 0.0001; CeC vs CeM, *p* = 0.0103; CeL vs CeM, *p* = 0.0021) and *oprm1* *+* *oxtr* *+* *oprk1* cells (one-way ANOVA, *F*_(2,12)_ = 19.00; *p* = 0.0002; CeC vs CeL, *p* = 0.0002; CeC vs CeM, *p* = 0.0659; CeL vs CeM, *p* = 0.0033). *p < 0.05, relative to CeC; ^☨^*p* < 0.05, relative to CeL.

The total cell population could be separated into nonexclusionary subsets of *oxtr*-expressing cells, *oprm1*-expressing cells, and *oprk1*-expressing cells (Table 3, Fig. 7). The central amygdala had 20,645 *oprm1*-expressing cells, 11,807 *oxtr*-expressing cells, and 7,568 *oprk1*-expressing cells. The number of each cell type (exclusionary populations) and subset (nonexclusionary population) across the central amygdala (CeL, CeC, and CeM) are reported in Table 2. Below, we report distributions of cell types across each subset. Figure 7 shows the subset of *oxtr*-expressing cells. Table 3 shows subsets of *oprm1*- and *oprk1*-expressing cells.

**Table 3.** Average distribution of *oprm1* and *oprk1* receptor-expressing cells

	Central amygdala	CeC	CeL	CeM
Proportion of *oprm1*-expressing cells that express:				
* *Proportion of *oprm1*-expressing cells that express *oxtr* (*oprm1* *+* *oxtr* only, *oprm1* *+* *oxtr* *+* *oprk1*)	52.8 ± 2.6%	54.8 ± 3.9%	58.1 ± 3.2%	50.0 ± 3.1%
* *Proportion of *oprm1*-expressing cells that express *oxtr* only (*oprm1* + *oxtr* only)	31.5 ± 1.9%	39.2 ± 2.2%	23.2 ± 1.2%	30.5± 3.2%
* *Proportion of *oprm1*-expressing cells that express *oprk1* (*oprm1* *+* *oprk1* only, *oprm1* *+* *oxtr* *+* *oprk1*)	34.0 ± 1.3%	27.1 ± 0.6%	47.1 ± 3.2%	32.8 ± 2.5%
* *Proportion of *oprm1*-expressing cells that express *oprk1* only (*oprm1* *+* *oprk1* only)	12.7 ± 1.2%	11.5 ± 1.5%	12.3 ± 1.4%	13.3 ± 1.5%
* *Proportion of *oprm1*-expressing cells that express *oxtr* and *oprk1* (*oprm1* *+* *oxtr* *+* *oprk1*)	21.3 ± 1.1%	15.6 ± 1.8%	34.8 ± 3.9%	19.4 ± 1.7%
Proportion of *oprk1*-expressing cells that express:				
* *Proportion of *oprk1*-expressing cells that express *oprm1* (*oprm1* *+* *oprk1* only, *oprm1* *+* *oxtr* *+* *oprk1*)	94.0 ± 0.9%	93.9 ± 1.3%	93.7 ± 1.8%	94.1 ± 2.3%
* *Proportion of *oprk1*-expressing cells that express *oprm1* only (*oprm1* *+* *oprk1 only*)	35.1 ± 3.1%	40.1 ± 5.5%	24.8 ± 3.1%	38.5 ± 3.9%
* *Proportion of *oprk1*-expressing cells that express *oxtr* (*oxtr* *+* *oprk1* only, *oprm1* *+* *oxtr* *+* *oprk1*)	61.4 ± 3.1%	55.2 ± 5.8%	71.6 ± 3.8%	58.8 ± 3.4%
* *Proportion of *oprk1*-expressing cells that express *oxtr* only (*oxtr* *+* *oprk1 only*)	2.5 ± 0.7%	1.5 ± 0.4%	2.7 ± 0.9%	3.2 ± 1.7%
* *Proportion of *oprk1*-expressing cells that express *oprm1* and *oxtr* (*oprm1* *+* *oxtr* *+* *oprk1*)	58.9 ± 2.5%	53.8 ± 5.6%	68.9 ± 4.5%	55.6 ± 2.1%

The data are expressed as mean ± standard error of the mean.

### Distribution of *oxtr*-expressing cells

Assessment of the subset of central amygdala cells that expressed *oxtr* revealed that most of these cells (93.4 ± 1.8%) expressed *oprm1* (regardless of *oprk1* colocalization). This was calculated by adding the number of cells that expressed *oxtr* and *oprm1* (i.e., *oprm1* *+* *oxtr* cells and *oprm1* *+* *oxtr* *+* *oprk1* cells), divided by the total number of cells that expressed *oxtr* (i.e., *oprm1* *+* *oxtr* cells, *oprm1* *+* *oxtr* *+* *oprk1* cells, *oxtr* *+* *oprk1* cells, and *oxtr*-only cells). We found that 37.8 ± 1.8% of *oxtr*-expressing cells expressed both *oprm1* and *oprk1* (i.e., *oprm1* *+* *oxtr* *+* *oprk1* cells).

The proportion of *oxtr*-expressing cells that colocalized *oprm1* and *oprk1* (i.e., *oprm1* *+* *oxtr* *+* *oprk1* cells) changed significantly across central amygdala subdivisions. The proportion of *oprm1* *+* *oxtr* *+* *oprk1* cells was higher in the CeL compared with the CeC (*p* = 0.0002) and CeM (*p* = 0.0033). The CeM was not significantly different from the CeC. However, we observed a trend toward the proportion of *oprm1* *+* *oxtr* *+* *oprk1* cells being more prominent in the CeM than in the CeC (*p* = 0.0659).

The proportion of *oxtr*-expressing cells that colocalized only *oprm1* (i.e., *oprm1* *+* *oxtr-*only cells) also changed significantly across central amygdala subdivisions. The proportion of *oprm1* *+* *oxtr*-only cells was lower in the CeL compared with the CeC (*p* < 0.0001) and CeM (*p* = 0.0021). The proportion of *oprm1* *+* *oxtr*-only cells was also lower in the CeM compared with the CeC (*p* = 0.0103).

### Distribution of *oprm1*- and *oprk1*-expressing cells

Assessment of the subset of central amygdala cells that expressed *oprm1* and *oprk1* revealed that 35.2 ± 1.7% of *oprm1*-expressing cells did not express *oxtr* or *oprk1*, whereas a majority (94.0 ± 0.9%) of *oprk1*-expressing cells expressed *oprm1*. The relative abundance of each subset supported this finding (Table 2). The density of each mRNA is shown in Figure 2. By dividing the nonexclusionary population and multiplying by 100 [i.e., the number of *oprm1*-expressing cells (20,645) divided by the number of *oprk1*-expressing cells (7,568) multiplied by 100], we found that *oprm1*-expressing cells were 2.7-fold more abundant than *oprk1*-expressing cells.

## Discussion

The present study demonstrated that a large proportion (43.8 ± 1.9%) of the total central amygdala cell population expresses both *oprm1* and *oxtr*, including those that express *oprk1*. Specifically, 40.0 ± 1.5% of cells that expressed *oprm1* and *oxtr* colocalized *oprk1*. We also evaluated all three subsets of *oxtr*-, *oprm1*-, and *oprk1*-expressing cells. We found that *oprm1* + *oxtr* cells comprised most of the *oxtr*-expressing cell subset (93.6 ± 1.8%), including those that coexpress *oprk1*. However, only 52.8 ± 2.6% of the *oprm1*-expressing cell subset colocalized *oxtr*. This subset-derived proportion difference can be explained by each mRNA's relative density and each cell type's density. The density of *oprm1* puncta was 2.6-fold higher than *oxtr* puncta. Similarly, the density of *oprm1*-expressing cells was 1.7-fold higher than *oxtr*-expressing cells. Similar to the *oxtr*-expressing cell subset, a majority of the *oprk1*-expressing cell subset colocalized *oprm1* (94.0 ± 0.9%), whereas a smaller proportion of *oprk1*-expressing cells colocalized *oxtr* (61.4 ± 3.1%). Notably, one-third of *oprm1*-expressing cells colocalized *oprk1* (34.0 ± 1.3%), and one-third of *oprm1*-expressing cells expressed no other transcript (35.2 ± 1.7%). Evaluating *oprm1* and *oprk1* mRNA density and cell density revealed that the relative density of *oprm1* puncta was 3.5-fold higher than *oprk1* puncta, and the density of *oprm1*-expressing cells was 2.7-fold higher than *oxtr*-expressing cells. We also found significant differences in cell-type distribution among the central amygdala subdivisions (CeC, CeL, and CeM) and across the rostrocaudal axis for cells that expressed *oprm1* and *oxtr*.

Our morphological data may provide a neuroanatomical basis for our previous behavioral finding that blocking opioid receptors can modulate oxytocin system function in the context of anxiety-like behaviors (Nisbett et al., 2024). The demonstration of cells that colocalize *oprm1* and *oxtr* suggests that this behavioral modulation could be mediated at least partially by μ-opioid and oxytocin receptors in the central amygdala. However, further behavioral and functional analysis is required to determine whether the central amygdala is necessary and sufficient to mediate this opioid system's modulation of oxytocin function.

**Figure 8.**
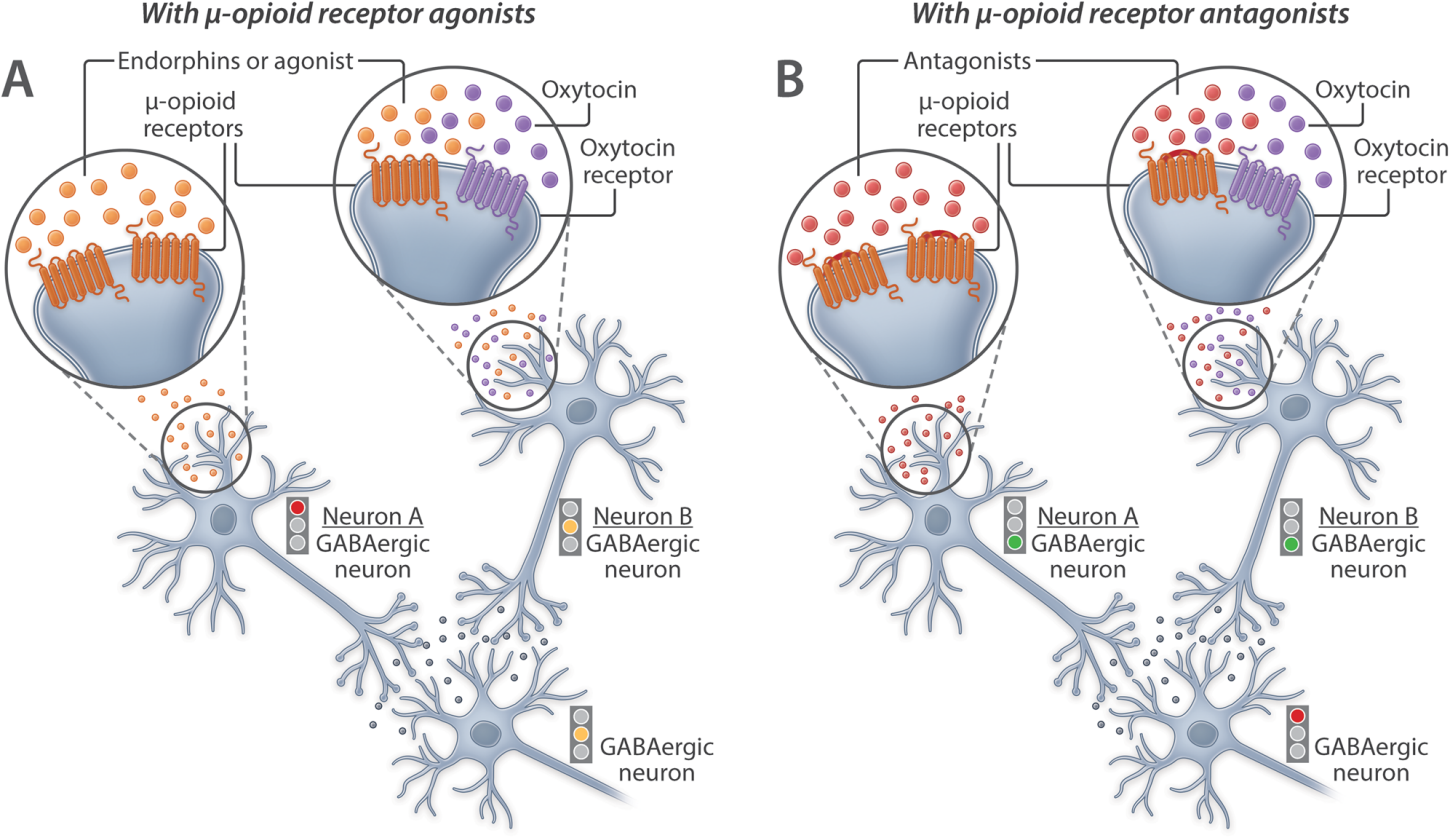
MOXI hypothesis. The present finding that most of *oxtr*-expressing cells colocalized *oprm1*, whereas a large proportion of *oprm1*-expressing cells did not colocalize *oxtr*, supports our hypothesis of how μ-opioid and oxytocin receptors may interact in the central amygdala. Given that the central amygdala is predominantly γ-aminobutyric acid (GABA)-ergic, we propose that μ-opioid receptor activation can inhibit GABA release from cells that express μ-opioid receptors (Neuron A) and cells that express both μ-opioid and oxytocin receptors (Neuron B). ***A***, Conversely, oxytocin receptor activation can promote GABA release. ***B***, As such, μ-opioid receptor antagonism may block the constitutive endorphin-mediated inhibition of GABAergic neurons, thereby disinhibiting μ-opioid receptor-expressing GABAergic neurons and enhancing oxytocin-induced GABAergic neuron stimulation and GABA release. Modified from Nisbett (2024).

Our previous behavioral data also demonstrated opposing effects of μ- and κ-opioid receptor antagonists on oxytocin function. We found that μ-opioid receptor antagonists potentiated the anxiolytic-like effect of oxytocin, whereas κ-opioid receptor antagonists inhibited the anxiolytic-like effect of oxytocin (Nisbett et al., 2024). The present observation that most *oxtr*-expressing cells colocalized *oprm1* (93.6 ± 1.8%) and that a large proportion of *oprm1*-expressing cells did not colocalize *oxtr* (39.4 ± 2.6%) informs our previous behavioral data and suggests that μ-opioid receptors may modulate oxytocin system function through actions at extrahypothalamic brain regions, such as the central amygdala. Thus, we suggest an additional hypothesis, namely, the μ- (Mu-) opioid × OXytocin receptor Interaction (MOXI) hypothesis (Nisbett, 2024). This hypothesis posits that stimulating μ-opioid receptors that colocalize or not with oxytocin receptors can increase the inhibitory tone of a converging neuron to prevent neurotransmitter release (Neuron A) and dampen the excitatory effect of oxytocin receptor stimulation (Neuron B; Fig. 8*A*). Conversely, blocking μ-opioid receptors can disinhibit neurons to allow neurotransmitter release (Neuron A) and/or augment the excitatory effect of oxytocin receptor stimulation (Neuron B; Fig. 8*B*).

The observed opposing effects of μ- and κ-opioid receptor antagonists in the context of emotion regulation are corroborated by previous studies. For example, the intracerebroventricular and intra-amygdalar administration of the selective μ-opioid receptor antagonist, CTOP, and selective κ-opioid receptor agonist, dynorphin A, similarly increased anxiety-like behavior of mice in the light/dark box (Narita et al., 2006). Similar opposing effects have been shown in models of nociception, pain, and drug dependence (Dal Monte et al., 2017; Head et al., 2021; Nisbett et al., 2024).

The primary explanation for opposing functions of μ- and κ-opioid receptors has been that both receptors are expressed on physiologically different neurons with opposing circuit functions (Pan, 1998). This has been hypothesized because μ- and κ-opioid receptors were found to couple G_i/o_ proteins similarly and have similar downstream effectors. However, our finding that most *oprk1*-expressing cells colocalized *oprm1* suggests an alternative parallel explanation for the opposing interaction between μ- and κ-opioid receptors. One possibility is that μ- and κ-opioid receptors heterodimerize, which can alter their function compared with their respective monomeric receptors (Jordan and Devi, 1999). However, this has not been supported by previous reports. A previous study by Jordan et al. demonstrated potential for μ- and δ-opiod receptor heterodimerization but not μ- and κ-opioid receptor heterodimerization (Jordan et al., 2000). Others have reported on the oligomerization of μ-, κ-, and δ-opioid receptors with other proteins (Jordan et al., 2000; Suzuki et al., 2002), suggesting that there is potential for protein–protein interaction to alter their functional properties.

As discussed above, the central amygdala can be divided into three parts: CeC, CeL, and CeM, based on immunohistochemical markers, morphology, and function (McDonald, 1982; Cassell et al., 1986; Jolkkonen and Pitkänen, 1998; Swanson and Petrovich, 1998; Pitkanen, 2000; Dong et al., 2001). Our finding that the distribution of cells in the CeC is more similar to the CeM than the CeL is surprising, especially considering previous reports that the CeC and CeL have similar projections and functionality (Petrovich and Swanson, 1997; Jolkkonen and Pitkänen, 1998; Bourgeais et al., 2001) and that both have different functionality than the CeM (Krettek and Price, 1977; Veening et al., 1984; Bourgeais et al., 2001; Dong et al., 2001). For example, the CeC and CeL have been shown to have few efferent projections and predominantly provide inputs to the CeM (Petrovich and Swanson, 1997; Jolkkonen and Pitkänen, 1998; Ciocchi et al., 2010), whereas the CeM projects extensively to other brain regions, including brainstem nuclei, the bed nucleus of the stria terminalis, and the lateral hypothalamus, to mediate physiological and behavioral responses to stressful stimuli.

This difference in cell-type distribution across subdivisions of the central amygdala can be explained by previous studies that categorized cell populations of the CeL (and CeC) based on electrophysiological properties and immunohistochemical markers (Cassell and Gray, 1989; Ciocchi et al., 2010; Haubensak et al., 2010). One population expresses protein kinase Cδ and comprises predominantly late-firing neurons (Li et al., 2013). They are referred to as CeL_OFF_ neurons because they respond to fear-conditioned stimuli with reduced activity (Ciocchi et al., 2010; Haubensak et al., 2010). The other population instead expresses somatostatin, which can be later firing or regular spiking (Li et al., 2013). They react to fear-conditioned stimuli with increased activity and are called CeL_ON_ (Ciocchi et al., 2010; Haubensak et al., 2010). They comprise a complex local network and have intrinsic rules (Haubensak et al., 2010; Hunt et al., 2017). For example, Haubensak et al. showed that neurons that do not express protein kinase Cδ can inhibit those that do, and neurons that express protein kinase Cδ are typically found in the CeM (Haubensak et al., 2010). In contrast, Hunt et al. showed that neuronal inhibition was more significant between neurons that expressed the same immunohistochemical markers (Hunt et al., 2017). Other gene markers that are used to identify cell types in the CeL include those for corticotropin-releasing factor and calcitonin receptor-like receptors (McCullough et al., 2018; O’Leary et al., 2022). Similar cell categories in the CeM were recently identified using immunohistochemical markers for nuclear receptor subfamily 2 group f member 2 (*Nr2f2*) and insulin gene enhancer protein (*Isl1*; O’Leary et al., 2022). Future studies are needed to determine whether *oprm1* *+* *oxtr* *+* *oprk1* cells, *oprm1-*only cells, *oprm1* *+* *oxtr*-only cells, and *oprm1* *+* *oprk1* cells belong to exclusively different cell types. For example, Haubensak et al. showed that 65% of CeL_OFF_ neurons express oxytocin receptors (Haubensak et al., 2010), and we found that a majority (94.0 ± 0.9%) of *oprk1*-expressing CeL cells colocalize *oprm1*, while a smaller proportion (68.9 ± 4.5%) of *oprk1*-expressing CeL cells colocalize *oprm1* and *oxtr*. As such, it is possible that CeL_OFF_ neurons express *oprk1* and *oprm1* with a large proportion of these cells also expressing *oprm1* and *oxtr*. As such, the presence of *oxtr* may change the functionality of CeL neurons. It is likely that similar mechanisms and functional differentiation occurs in the CeM; however, further research on this subregion is required to aid further speculation.

Herein, we also demonstrated differences in cell-type distribution across the rostrocaudal axis. One explanation for these differences is that similar to the cell types that were identified in our study, the distribution of immunohistochemical cell markers also significantly changes across the rostrocaudal axis (McCullough et al., 2018). McCullough et al. identified a distribution of six genetic markers that change significantly across the rostrocaudal axis. These include somatostatin, neurotensin, corticotropin-releasing factor, tachykinin 2, protein kinase Cδ, and the dopamine D_2_ receptor. Changes in the rostrocaudal axis are also different for the central amygdala subdivisions. The observed differences in cell-type distribution across the rostrocaudal axis may also correlate with efferent nuclei. Although not yet reported for the central amygdala, the medial amygdala has differential projections across the rostrocaudal axis (Gomez and Newman, 1992). Importantly, however, the medial amygdala is also sexually dimorphic (Cooke and Woolley, 2005; Bergan et al., 2014). Each cell type may be representative of a particular category of projecting neurons (defined by their efferent regions), which likely changes across the rostrocaudal axis and central amygdala subdivisions.

One caveat of the current findings is that mRNA expression does not necessarily correlate with protein expression (Mehra et al., 2003). Positive mRNA expression indicates that a cell can transcribe a particular protein; however, protein expression may be silent or more or less densely expressed than the respective mRNA expression. Future studies should measure protein expression or other proxies of protein expression (e.g., radioligand–receptor binding and positron emission tomography studies) in stress- and drug-naive animals to estimate the correlation between mRNA and protein expression. Advances beyond the present study should also include an investigation of how μ-opioid, κ-opioid, and oxytocin receptor expression changes with a broad range of emotional and physiological states (e.g., stress exposure and drug withdrawal).

In conclusion, we demonstrated three previously undescribed populations of cells in the brain. These include cells that coexpress *oprm1* and *oxtr*; cells that coexpress *oprm1*, *oxtr*, and *oprk1*; and cells that coexpress *oprm1* and *oprk1*. We hypothesize that *oprm1* + *oxtr* cells may play a role in the previously described interaction between oxytocin receptor agonists and μ-opioid receptor antagonists (Nisbett et al., 2024) and provide morphological support for an interaction between μ-opioid and oxytocin receptors within central amygdala cells and its broader network (Nisbett, 2024). Further studies are needed to understand how μ- and κ-opioid receptor activity affects oxytocin system function.

## Synthesis

Reviewing Editor: Timothy Jarome, Virginia Polytechnic Institute and State University Decisions are customarily a result of the Reviewing Editor and the peer reviewers coming together and discussing their recommendations until a consensus is reached. When revisions are invited, a fact-based synthesis statement explaining their decision and outlining what is needed to prepare a revision will be listed below. The following reviewer(s) agreed to reveal their identity: NONE. Note: If this manuscript was transferred from JNeurosci and a decision was made to accept the manuscript without peer review, a brief statement to this effect will instead be what is listed below.

Both reviewers agreed that the study was novel, well designed and interpreted. However, several concerns need addressed before this is ready for publication:

1. The probes used seem to have a commercial origin and the company that provides these probes has many unique probes for different receptors. Could the authors explain how the probes have been validated and how they chose among the different available probes?

2. The authors made a beautiful scheme about the Moxi hypothesis working on separate neurons modulating effects of OT inhibition. They then hypothesize how kappa opioid receptor can hetermodimerize with mu opoid receptor to explain the opposite effects of these two opioid receptors. Neither hypothesis is further tested and rather speculative. Considering this, can the authors integrate BOTH hypotheses into the scheme that they put forward in figure 8?

3. In addition, they show differential localization of the different receptor subtypes in different regions. Can they integrate these two findings in a model that includes CeL, CeM and CeC, taking into account how these receptors have been already found to be differentially expressed in these subregions in other studies? (e.g. Haubensak et al., 2010).

4. Related, when the authors reference their model (Figure 8) in the narrative they refer to Figure 6A and 6B. Also, they refer to Neuron A and Neuron B. However, there are no labels in the schematic of Neuron A and B. Overall, the schematic is a bit confusing and should be improved to make a clearer impact of what their hypothesis is.

5. It was unclear if data analysis included cells with expression of none of the receptors studied.

6. When referring to data in Fig 5 and Fig 6 (etc), please include references to the figures in the description of the data so that it is easier to follow the narrative.

7. Explain in a clear way what is meant by exclusionary versus non-exclusionary criteria.

8. In table 2B, how can oprk1 expressing cells express "opmr1 only" in column two. Did you mean "oprk1 only"

9. In Figures 5 and 6 there should be a legend so that the reader can immediately sort out which color belings to each group/category. You should not expect a reader to follow your color pattern explicitly.

10. Please add page and line number
