# Supplementary figures and images for: Neuronal Colocalization of μ-Opioid Receptor, κ-Opioid Receptor, and Oxytocin Receptor mRNA in the Central Nucleus of the Amygdala in Male and Female Mice
Source: eNeuro. 2025 Sep 5;12(9):ENEURO.0059-25.2025. doi: 10.1523/ENEURO.0059-25.2025 (PMC12439755; doi:10.1523/ENEURO.0059-25.2025)

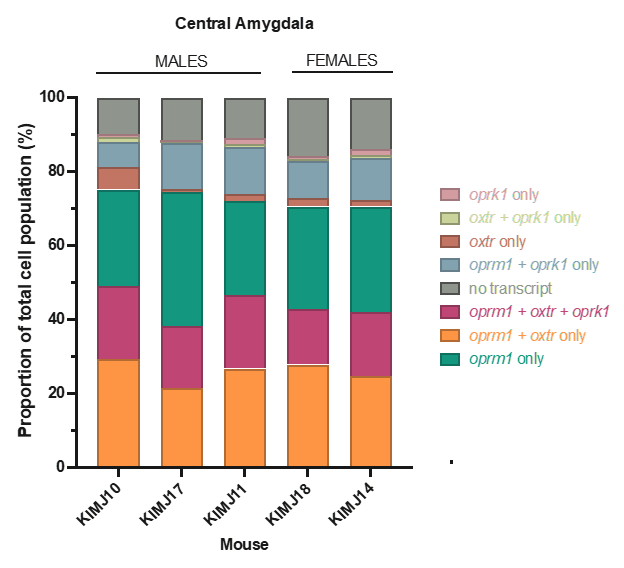

Supplement: Figure 3-1 — The distribution of various cell types across each experimental animal is similar. Download Figure 3-1, TIF file. [file eneuro-12-ENEURO.0059-25.2025-s002.tif]

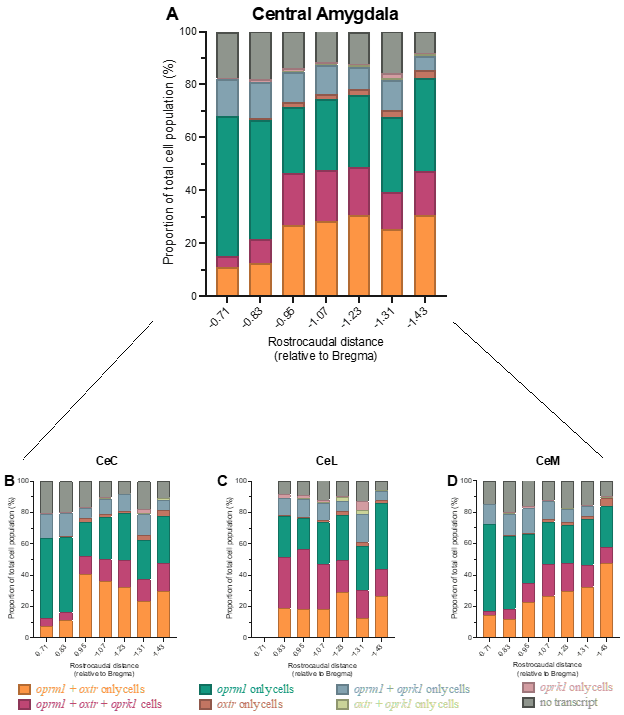

Supplement: Figure 6-1 — Distribution of each cell type across the rostrocaudal axis in the central amygdala (A) and each subdivision: CeC (B), CeL (C), and CeM (D). In the central amygdala, the mean cell distribution ranges were the following: oprm1 + oxtr-only (7.6-35.7%), oprm1 + oxtr + oprk1 (3.9-30.7%), oprm1-only (17.6-53.1%), oxtr-only (0-18.1%), oprm1 + oprk1-only (5.4-24.5%), oxtr + oprk1 (0.1-4.7%), oprk1-only (0.3-4.4%), no transcript (5.8-20.8%) (A). In the CeC, the mean cell distribution ranges were the following oprm1 + oxtr-only (7.6-40.9%), oprm1 + oxtr + oprk1 (5.2-17.9%), oprm1-only (21.6-50.9%), oxtr-only (0-4.0%), oprm1 + oprk1-only (6.0-15.1%), oxtr + oprk1 (0.0-1.1%), oprk1-only (0.3-2.6%), no transcript (7.8-20.6%) (B). In the CeL, the mean cell distribution ranges were the following: oprm1 + oxtr-only (6.1-37.0%), oprm1 + oxtr + oprk1 (12.4-55.7%), oprm1-only (14.8-42.4%), oxtr-only (0.3-4.0%), oprm1 + oprk1-only (4.7-27.8%), oxtr + oprk1 (0.0-5.8%), oprk1-only (0.0-9.4%), no transcript (2.9-17.1%). Note that the CeL was not present at anterior/posterior coordinate -0.71 relative to bregma (C). In the CeM, the mean cell distribution ranges were the follow: oprm1 + oxtr-only (12.0-47.4%), oprm1 + oxtr + oprk1 (2.8-20.2%), oprm1-only (24.1-55.2%), oxtr-only (0.0-1.7%), oprm1 + oprk1-only (0.0-15.4%), oxtr + oprk1 (0.0-0.6%), oprk1-only (0.3-1.0%), no transcript (9.7-19.8% (D). Download Figure 6-1, TIF file. [file eneuro-12-ENEURO.0059-25.2025-s003.tif]
